# Peripheral brain-derived neurotrophic factor in autism spectrum disorder: a systematic review and meta-analysis

**DOI:** 10.1038/srep31241

**Published:** 2016-08-10

**Authors:** Zhen Zheng, Li Zhang, Tingting Zhu, Jichong Huang, Yi Qu, Dezhi Mu

**Affiliations:** 1 Department of Pediatrics, West China Second University Hospital, Sichuan University, Chengdu 610041, China; 2 Key Laboratory of Obstetric & Gynecologic and Pediatric Diseases and Birth Defects of Ministry of Education, Sichuan University, Chengdu 610041, China; 3 Department of Pediatrics and Neurology, University of California, San Francisco, San Francisco, CA94143, USA

## Abstract

Brain-derived neurotrophic factor (BDNF) regulates neuronal survival and growth and promotes synaptic plasticity. Recently, researchers have begun to explore the relationship between peripheral BDNF levels and autism spectrum disorder (ASD), but the findings are inconsistent. We undertook the first systematic review and meta-analysis of studies examining peripheral BDNF levels in ASD compared with healthy controls. The PubMed, Embase, and Cochrane Library databases were searched for studies published before February 2016. Fourteen studies involving 2,707 participants and 1,131 incident cases were included. The meta-analysis provided evidence of higher peripheral BDNF levels in ASD compared with controls [standardized mean difference (SMD) = 0.63, 95% confidence interval (95% CI) = 0.18–1.08; *P* = 0.006]. Subgroup analyses revealed higher BDNF levels in ASD compared with controls for both serum [SMD = 0.58, 95% CI = 0.11–1.04; *P* = 0.02] and plasma [SMD = 1.27, 95% CI = 0.92–1.61; *P* < 0.001]. Studies of childhood yielded similar cumulative effect size [SMD = 0.78, 95% CI = 0.31–1.26; *P* = 0.001], while this was not true for the studies of adulthood [SMD = 0.04, 95% CI = −1.72–1.80; *P* = 0.97]. This meta-analysis suggests that peripheral BDNF levels are a potential biomarker of ASD.

Autism spectrum disorder (ASD) is a neurodevelopmental disorder characterized by abnormalities in social interaction, impairment in language and communication, restrictive or repetitive interests, and stereotyped behaviours and movements^1^. ASD includes autistic disorder, Asperger syndrome, and pervasive developmental disorder not otherwise specified^1^. Over the last decade, the prevalence of ASD has been reported as 11.3 per 1,000 with a male-to-female ratio of 3–4:1^2^. There is growing evidence that ASD may be influenced by genetic, neurological, environmental and immunological factors^3^,^4^. However, the underlying mechanism of ASD has not yet been identified. Behavioural abnormalities are not evident until approximately 12–18 months of age^5^,^6^. Furthermore, ASD individuals vary enormously in clinical manifestation, severity, developmental trajectory, and treatment response. This complexity has propelled an intensive search to identify biomarkers to aid clinicians in achieving earlier diagnoses and in predicting treatment response^7^.

Brain-derived neurotrophic factor (BDNF) is a small protein found throughout the central nervous system (CNS) and peripheral blood. BDNF plays an important regulatory function in cell proliferation, migration, and survival during neurodevelopment and can also modulate axonal and dendritic outgrowth, synapse formation, neurotransmitter release and other neuroplastic processes^8^,^9^. Converging lines of evidence implicate the role of BDNF in the pathophysiology of ASD. It is reported that the concentration of BDNF in serum and the CNS are closely correlated in rats^10^. However, evidence of such a correlation in humans is still lacking. Therefore, whether altered BDNF values in the periphery reflect altered BDNF levels in the human CNS requires further investigation. Scientists assume that peripheral BDNF levels mirror and indirectly reflect BDNF levels in the brain^11^. This indirect measure of BDNF levels is much easier to acquire than direct assessments of BDNF levels in the CNS. For this reason, the concentration of BDNF in peripheral blood may be a useful potential biological marker for ASD.

The concept of the “periphery as a window to the brain” has led to an ever-increasing number of clinical studies assessing peripheral BDNF levels in ASD. However, reports of peripheral BDNF levels in ASD are inconsistent. Some studies have reported that serum BDNF is significantly reduced in ASD compared with healthy controls^12^,^13^, while other studies have reported higher serum BDNF levels in children with ASD compared with controls^14^,^15^,^16^. Similarly, two studies that have examined BDNF levels in neonatal specimens from individuals later diagnosed with ASD have yielded inconsistent results^17^,^18^.

Thus, we undertook a systematic review of studies assessing peripheral BDNF levels in ASD and controls, followed by a series of meta-analyses to provide an overall estimate of the effect size and between-study heterogeneity of the association between peripheral BDNF levels and ASD.

## Results

### Literature search

**Figure 1:**
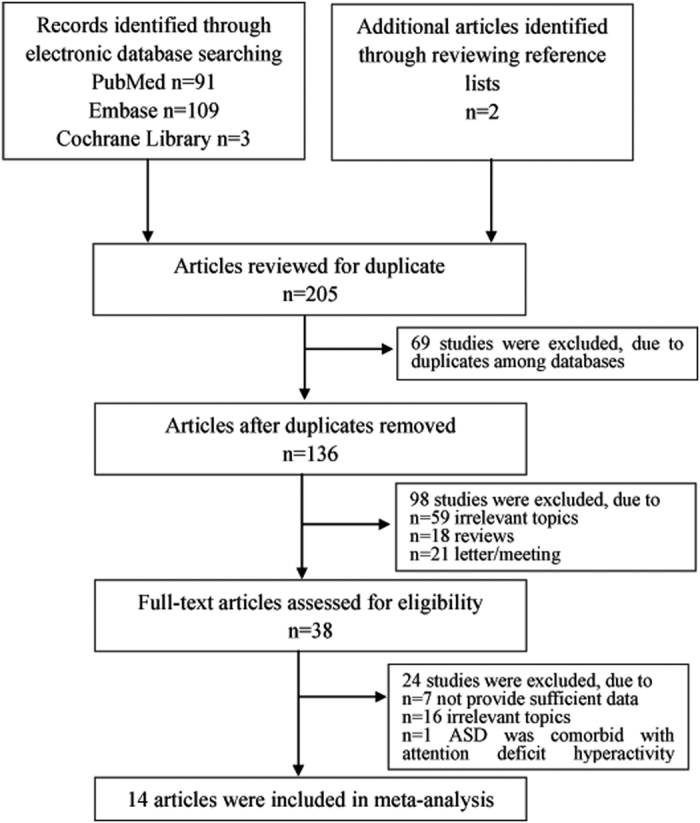
Flow chart of the study selection process to identify studies eligible for the systematic review.

The initial search yielded a total of 205 citations: 91 from PubMed, 109 from Embase, 3 from Cochrane Library and 2 from reviewing references. After excluding 69 duplicate studies, 59 with irrelevant topics, 18 reviews and 21 letters/meetings, 38 studies of peripheral BDNF levels in ASD were identified and subjected to detailed evaluation. Subsequently, 16 studies were excluded due to irrelevant outcomes. Seven reports that lacked sufficient data (raw data mean and standard deviation (SD) or median and interquartile range (IQR)) were also excluded. One report was excluded because the ASD was comorbid with attention deficit hyperactivity disorder. Finally, 14 studies, including 2,707 participants and 1,131 incident cases, fulfilled all of the inclusion criteria and were included in the meta-analysis. A detailed flow chart of the search and selection process is presented in Fig. 1.

### Study characteristics

The characteristics of the fourteen selected studies are presented in Table 1. All of the studies were published between 2001 and 2016. 14 studies including 2,707 participants and 1,131 incident cases were included in this meta-analysis. Three studies were conducted in the United States^18^,^19^,^20^, three in China^16^,^21^,^22^, three in Japan^13^,^23^,^24^, one in Denmark^17^, one in Ireland^14^, one in Saudi Arabia^25^, one in India^26^, and one in Italy^15^. The sample sizes varied widely, ranging from 18^13^,^24^ to 359^17^ ASD individuals and from 16^24^ to 741^17^ controls. Similarly, the mean age of the ASD and control individuals varied broadly and ranged from 0^17^,^18^,^19^,^20^ to 22.2 ± 2.2^13^ years old. The systematic review identified two different biomaterials used for BDNF assays: serum^13^,^15^,^16^,^17^,^18^,^19^,^20^,^21^,^22^,^23^,^24^,^25^,^26^ and plasma^14^. Moreover, 11 studies assessed the BDNF levels using enzyme-linked immunosorbent assay (ELISA) as the analytical procedure^13^,^14^,^15^,^16^,^20^,^21^,^22^,^23^,^24^,^25^,^26^, while 3 adopted Luminex^17^,^19^,^20^ and 1 used recycling immunoaffinity chromatography (RIAC)^18^.

### Meta-analysis of peripheral BDNF levels in ASD

**Figure 2:**
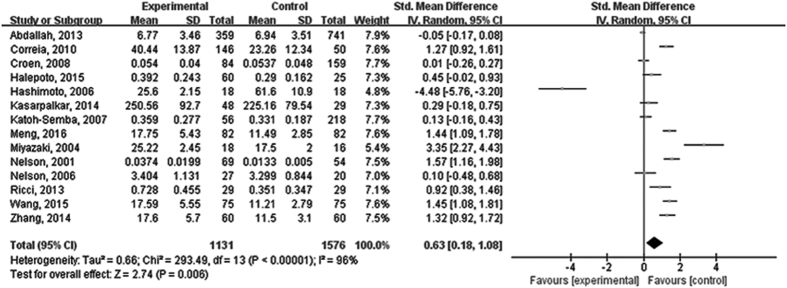
Forest plot of random-effect between-group meta-analysis of peripheral BDNF levels in persons with ASD and healthy controls. ASD: autism spectrum disorder.

Based on estimates pooled from 14 studies, a significantly higher level of BDNF was found in ASD compared with controls [standardized mean difference (SMD) = 0.63, 95% CI = 0.18–1.08; *P* = 0.006)]. However, there was significant statistical heterogeneity across studies (I^2^ = 96%, *P* < 0.001) (Fig. 2).

### Quality evaluation

The results of the quality assessment of the included studies are shown in Table 2. Fourteen studies were of high quality, with an average score of 7.4.

### Publication bias

**Figure 3:**
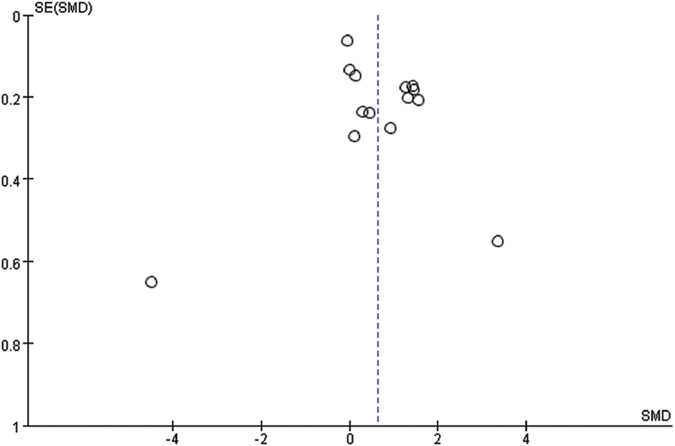
Funnel plot of random-effect between-group meta-analysis of peripheral BDNF levels in persons with ASD and healthy controls. ASD: autism spectrum disorder.

The funnel plot was symmetric (Fig. 3). Moreover, Begg’s and Egger’s tests did not reveal significant evidence of publication bias among the included studies (Begg’s test, *P* = 1.000; Egger’s test, *P* = 0.156).

### Subgroup analysis

Of the fourteen studies included in this meta-analysis, thirteen described BDNF levels assessed in serum. One study reported result assessed in plasma. Higher BDNF levels were found in ASD compared with controls in both subgroups of studies. The pooled SMD was 0.58 (95% CI = 0.11–1.04, *P* = 0.02) for BDNF levels assessed in serum and 1.27 (95% CI = 0.92–1.61, *P* < 0.001) for BDNF levels assessed in plasma (Table 3).

We conducted a meta-analysis of the five studies of ASD and the nine studies of autism. The pooled SMD was 0.64 (95% CI = 0.1–1.19, *P* = 0.02) for the studies of ASD and 0.55 (95% CI = −0.03–1.13, *P* = 0.06) for the studies of autism (Table 3).

We conducted a meta-analysis of the ten studies of childhood and the four studies of adulthood. The pooled SMD was 0.78 (95% CI = 0.31–1.26, *P* = 0.001) for the studies of childhood and 0.04 (95% CI = −1.72–1.80, *P* = 0.97) for the studies of adulthood (Table 3).

We also conducted a meta-analysis of the studies based on the analytic technology used: Luminex, ELISA or RIAC. The effect size of the difference in BDNF levels measured in ASD and controls when these different analytical technologies were applied was 0.66 (95% CI = 0.12–1.2, *P* = 0.02) for ELISA, −0.03 (95% CI = −0.14–0.08, *P* = 0.56) for Luminex, and 1.57 (95% CI = 1.16–1.98, *P* < 0.001) for RIAC (Table 3).

Meta-analysis of the studies based on both subject age and the analytical technology used showed that the pooled SMD was 0.93 (95% CI = 0.54–1.33, *P* < 0.001) for the studies of childhood and 0.04 (95% CI = −1.72–1.8, *P* = 0.97) for the studies of adulthood when applying ELISA. The pooled SMD was −0.03 (95% CI = −0.14–0.08, *P* = 0.56) for the studies of childhood and used Luminex as the analytical method. The pooled SMD was 1.57 (95% CI = 1.16–1.98, *P* < 0.001) for the study of childhood and employed RIAC as the analytical method (Table 3).

### Meta-regression analysis

We performed meta-regression analyses in an exploratory attempt to identify the sources of heterogeneity between the studies and the effect of moderators. Using univariable meta-regression models, we found a positive relationship between gender and BDNF levels (slope = 0.06, 95% CI = 0.01–0.11; *P* = 0.024). There was no relationship between the mean age, study design or confounders adjustment and BDNF levels (Table 4).

### Sensitivity analysis

The influence of each study on the overall estimate was assessed by removing studies one by one and comparing the pooled estimate from the remaining thirteen studies to the pooled estimate from all fourteen studies. The results revealed higher peripheral BDNF levels in ASD compared with controls in all 14 analyses, indicating that the removal of any one study would not alter the overall results.

## Discussion

Over the fourteen studies, 2,707 participants and 1,131 incident cases were included in this meta-analysis. A random-effect model was used to compute the pooled estimates because there was significant between-study heterogeneity. The pooled SMD indicated that the peripheral BDNF level was higher in ASD compared to the controls. Sensitivity analysis showed that the pooled results were robust. The symmetrical funnel plot and the results of Begg’s and Egger’s tests also suggested the lack of significant publication bias.

BDNF, which is the most abundant neurotrophin in the CNS, can cross the blood-brain barrier. Therefore, its levels in serum and plasma are highly correlated with the levels in cerebrospinal fluid (r = 0.8)^10^,^27^. Studies have shown that BDNF protein levels in serum and the brain are similar in developmental rats, with a positive correlation between serum and cortical BDNF levels^10^. In addition, Klein *et al*.^28^ reported a significant positive correlation between whole blood and hippocampal BDNF levels in rats. Furthermore, they demonstrated that blood and plasma BDNF levels also reflected brain-tissue BDNF levels in rats and pigs. However, BDNF levels in blood are undetectable in other species, such as mice. Additionally, evidence of such a correlation in humans is still lacking. Therefore, whether altered BDNF values in the periphery reflect altered BDNF levels in the human CNS requires further investigation.

Interestingly, we found that peripheral BDNF levels were higher in the ASD samples from childhood but not from adulthood. Evidence from animal models of ASD suggests that BDNF levels increase in the foetal brain^29^,^30^. In the ASD model of sodium valproate (VPA) exposure in utero, VPA administration has been shown to increase BDNF protein levels in the feotal mouse brain 5- to 6-fold *in vitro* and *in vivo*^29^. Higher BDNF expression in the foetal brain was also demonstrated in the BTBR T+tf/J ASD model^30^. BDNF hyperactivity during early life may play an aetiological role in ASD. Early BDNF hyperactivity could result in the overgrowth of brain tissue, which is found in many ASD children^31^. Increased BDNF levels in children with ASD may reflect a regional compensatory mechanism in response to late brain maturation^32^. Therefore, accurately detecting BDNF levels is important to analyse the brain development in children with ASD.

Different analytical technologies for the assessment of BDNF were used across the studies. Four studies of adulthood and seven studies of childhood used ELISA to analyse BDNF levels. BDNF levels were found to be significantly increased in the childhood studies but not in the adulthood studies, further demonstrating the difference in peripheral BDNF levels between the age groups. Furthermore, subgroup analyses revealed that BDNF levels were significantly higher in children with ASD compared with controls when the levels were measured through ELISA and RIAC, but not when they were measured using Luminex. Therefore, the ELISA and RIAC assays may have been more sensitive than the Luminex assay in detecting BDNF levels in the included studies.

Significant heterogeneity was found in this analysis. To clarity the sources of heterogeneity and make a more comprehensive analysis, we performed the subgroup analyses and meta-regression. We found that three different factors contributed to the heterogeneity. First, the positive SMD is significantly higher in plasma-based samples than that in serum-based samples (Table 3). This difference may due to the difference of clotting factors between plasma and serum. Second, that different analytic technologies used in this study may also contribute to heterogeneity (Table 3). Finally, the gender factor especially for the percentage of male was also one of the reasons for the heterogeneity (Table 4).

Our study had some advantages. First, this is the first comprehensive meta-analysis conducted to assess the association between peripheral BDNF levels and ASD. Second, the sensitivity analysis indicated that the removal of individual studies did not alter the final results, which increased the robustness of the conclusions of this analysis. Third, no significant publication bias was detected, suggesting that the results are reliable.

However, our meta-analysis also had some limitations. Firstly, detailed information regarding medication use was not provided in some studies. Medication could have influenced peripheral BDNF levels in ASD. Thus, future research should take into consideration the possible effect of medication on peripheral BDNF levels. Secondly, the potential correlation between the severity of ASD and BDNF levels was not assessed because few studies have analysed the relationship between the clinical severity of ASD and BDNF levels. The association between the severity of ASD and BDNF should be evaluated in future studies. Thirdly, the meta-analysis of BDNF levels in ASD generated a pooled result that largely originated from cross-sectional studies. Therefore, we cannot draw any conclusions about causality. We do not know if an increase in BDNF levels is a cause or consequence of ASD development. Fourthly, there was significant heterogeneity in this analysis, which may have affected the precision of the overall results.

In conclusion, this meta-analysis indicated that peripheral BDNF levels are higher in ASD compared with controls, suggesting that peripheral BDNF levels may serve as a potential biomarker for the diagnosis of ASD. Future studies need to clarify the influence of medication, the causal nature of the relationship and the association between the severity of clinical ASD symptoms and BDNF levels.

## Methods

### Literature search

Two authors searched the PubMed, Embase and Cochrane Library databases for relevant articles published before February 2016 using both Medical Subject Heading (MeSH) terms and the free text terms “ASD,” “autism,” “autism spectrum disorder,” “autistic disorder,” “Asperger syndrome,” “pervasive developmental disorder,” and “BDNF,” “brain-derived neurotrophic factor,” and “peripheral,” “levels,” “serum,” “plasma,” “urine,” “saliva,” “blood,” “platelets,” “cerebrospinal fluid,” “red blood cells.” In addition, the references of the included articles and previous meta-analyses were manually searched to identify additional studies.

We restricted the search to human studies published in English. The titles and abstracts of the retrieved studies were reviewed to exclude studies that were clearly irrelevant. Then, two authors independently read the full text of the remaining studies to assess their eligibility according to the inclusion criteria. Disagreements about the inclusion/exclusion of a study were resolved by a third author, who independently examined the studies, and consensus was reached.

### Study selection

Studies were eligible for analysis if they met all of the following criteria: (1) they were about the association between peripheral BDNF levels and ASD *in vivo*; and (2) they provided the raw data mean and SD or median and IQR.

The following types of studies were excluded: (1) reviews, case reports, case-only studies, animal studies, and simple commentaries; (2) overlapping publications; (3) publications lacking measures of peripheral BDNF levels, including pharmacological, genetic, brain imaging, and post-mortem studies; (4) studies in which ASD was comorbid with other conditions; and (5) studies that showed BDNF levels in dot plot and histogram format but did not provide numerical results.

### Data extraction

Two authors extracted data from the included articles, with particular regard to the following: first author’s name, publication year, country of region, number of cases and controls, age of subjects (mean ± SD), percentage of females and males, analytical technology employed, biomaterial evaluated, BDNF level (mean ± SD), unit of measure and adjusted confounders. BDNF levels were measured from serum and plasma, and different units of measurement were used across studies (ng/ml or pg/ml). Therefore, we reported all BDNF levels in ng/ml (1 ng/ml = 1000 pg/ml). If the data were presented using the median (IQR) format, then the formula “IQR/1.35” was used to calculate SD. If participants overlapped between studies, the study with the largest sample size was included in the meta-analysis.

### Quality evaluation

Two authors independently assessed the quality of each included study using the Newcastle-Ottawa Quality Assessment Scale (NOS) to determine the quality of selection, comparability, exposure, and outcome of study participants, with a maximum score of 9 points. We divided the study quality into three categories: (1) high quality (scored 7–9); (2) moderate quality (scored 4–6); and (3) low quality (scored 0–3). Disagreements were resolved through mutual discussion.

### Statistical analysis

The SMD was used to assess the association between peripheral BDNF levels and ASD. We pooled the SMD across studies using the Mantel-Haenszel formula (fixed-effect model) or the DerSimonian-Laird formula (random-effect model). A fixed-effect model was chosen when heterogeneity was low; otherwise, a random-effect model was adopted. Across-study heterogeneity was evaluated using the I^2^ and Q statistics; these statistics provide a quantitative measure of inconsistency across studies, with suggested thresholds for low (25–50%), moderate (50–75%) and high (>75%) heterogeneity. The Q statistic was considered significant if *P* < 0.1, and I^2^ > 50% indicated high heterogeneity. The results of the analyses are shown in forest plots.

Potential publication bias was assessed via visual inspection of the funnel plot. Begg’s and Egger’s tests were used to estimate the severity of publication bias, with *P* < 0.05 considered statistically significant.

We analysed subgroups of studies to examine the source of potential heterogeneity based on the biomaterial used (serum or plasma), condition (ASD or autism) and subject age (childhood < 18 years old or adulthood ≥18 years old). The analytical technologies employed included Luminex, ELISA and RIAC.

Unrestricted maximum likelihood random effects meta-regressions of effect size were performed with mean age, gender (% male), study design and confounder adjustment as moderators to determine whether these covariates influenced the effect size.

We carried out the sensitivity analysis by removing studies one by one and comparing the SMD of the remaining studies to the SMD for all studies. Statistical analysis was performed using Stata 12.0 (Stata Corp, College Station, Texas, USA) and Cochrane Collaboration Review Manager 5.1.2 (Cochrane Collaboration, Oxford, UK) software.

## Additional Information

**How to cite this article**: Zheng, Z. *et al*. Peripheral brain-derived neurotrophic factor in autism spectrum disorder: a systematic review and meta-analysis. *Sci. Rep*. **6**, 31241; doi: 10.1038/srep31241 (2016).

## Figures and Tables

**Table 1:** Characteristics of the fourteen studies included in the meta-analysis.

Author Year	Country	Sample size ASD controls	Age Mean ± SD (range) ASD controls	Sex (F/M) ASD controls	Analytical technology	Biomaterial	BDNF Mean ± SD ASD controls	Unit of measure	Adjusted founders
Abdallah^17^	Denmark	359741	00	68/291146/595	Luminex	Serum	6.77 ± 3.466.94 ± 3.51	ng/ml	Age, gender
Correia^14^	Ireland	14650	7.17.5	nr	ELISA	Plasma	40.44 ± 13.8723.26 ± 12.34	ng/ml	nr
Croen^19^	America	84159	00	nr	Luminex	Serum	0.0541 ± 0.0400.0537 ± 0.048	ng/ml	Age, gender
Halepoto^25^	Saudi Arabia	6025	6 ± 1.727.04 ± 1.74	nr	ELISA	Serum	0.392 ± 0.2430.290 ± 0.162	ng/ml	Age, gender
Hashimoto^13^	Japan	1818	21.2 ± 2.122.2 ± 2.2	0/180/18	ELISA	Serum	25.6 ± 2.1561.6 ± 10.9	ng/ml	Age
Kasarpalkar^26^	India	4829	7.47.4	nr	ELISA	Serum	250.56 ± 92.7225.16 ± 79.54	ng/ml	Age
Katoh-Semba^23^	Japan	56218	<60<60	nr	ELISA	Serum	0.359 ± 0.2770.331 ± 0.187	ng/ml	Age
Miyazaki^24^	Japan	1816	7.6 ± 6.123.3 ± 0.9	1/1711/5	ELISA	Serum	25.22 ± 2.4517.5 ± 2.00	ng/ml	nr
Nelson^18^	America	6954	00	8/6127/27	RIAC	Serum	0.0374 ± 0.01990.0133 ± 0.005	ng/ml	nr
Nelson^20^	America	2720	00	nr	Luminex and ELISA	Serum	3.404 ± 1.1313.299 ± 0.844	ng/ml	Mean gestational age, birth weight
Ricci^15^	Italy	2929	2–212–21	2/272/27	ELISA	Serum	0.728 ± 0.4550.351 ± 0.347	ng/ml	Age, gender
Wang^16^	China	7575	4.0 ± 1.254.0 ± 1.25	13/6213/62	ELISA	Serum	17.59 ± 5.5511.21 ± 2.79	ng/ml	Age, gender
Zhang^21^	China	6060	3.78 ± 1.223.78 ± 1.22	12/4812/48	ELISA	Serum	17.6 ± 5.711.5 ± 3.1	ng/ml	Age, gender
Meng^22^	China	8282	4.02 ± 1.274.02 ± 1.27	17/6517/65	ELISA	Serum	17.75 ± 5.4311.49 ± 2.85	ng/ml	Age, gender

ASD: autism spectrum disorder; nr: not reported; F/M: female/male; RIAC: recycling immunoaffinity chromatography; ELISA: enzyme-linked immunosorbent assay.

**Table 2:** Quality assessment of the included studies based on the Newcastle–Ottawa Scale.

Publication year	Study design	Selection	Comparability	Exposure/Outcome	Total scores
Abdallah^17^	Cross-section	★★★★	★★	★★	8
Correia^14^	Cross-section	★★★	★	★★	6
Croen^19^	Case-control	★★★★	★★	★★	8
Halepoto^25^	Cross-section	★★★★	★★	★★	8
Hashimoto^13^	Cross-section	★★★	★★	★★	7
Kasarpalkar^26^	Cross-section	★★★★	★★	★★	8
Katoh-Semba^23^	Cross-section	★★★	★★	★★	7
Miyazaki^24^	Cross-section	★★★★	★	★★	7
Nelson^18^	Case-control	★★★★	★	★★	7
Nelson^20^	Case-control	★★★★	★★	★★	8
Ricci^15^	Cross-section	★★★	★★	★★	7
Wang^16^	Cross-section	★★★★	★★	★★	8
Zhang^21^	Cross-section	★★★★	★★	★★	8
Meng^22^	Cross-section	★★★	★★	★★	7

**Table 3:** Summary results of peripheral BDNF levels in persons with ASD and healthy controls.

Variables	No. of comparisions	No. of subjects		Meta-analysis				Heterogeneity		Test for subgroup differences	
		ASD	Controls	SMD	95% CI		P-value	I2	P-value	I2	P-value
Biomaterial											
Serum	13	985	1526	0.58	0.11	1.04	0.02	96	<0.001	81.6	0.02
Plasma	1	146	50	1.27	0.92	1.61	<0.001	Not applicable	Not applicable		
Condition											
ASD	5	538	974	0.64	0.1	1.19	0.02	92	<0.001	0	0.82
autism	9	593	602	0.55	−0.03	1.13	0.06	94	<0.001		
Subject age											
Childhood	10	1010	1295	0.78	0.31	1.26	0.001	96	<0.001	0	0.42
Adulthood	4	121	281	0.04	−1.72	1.8	0.97	97	<0.001		
Analytical technology											
ELISA	11	619	622	0.66	0.12	1.2	0.02	94	<0.001	96.6	<0.001
Luminex	3	470	920	−0.03	−0.14	0.08	0.56	0	0.84		
RIAC	1	69	54	1.57	1.16	1.98	<0.001	Not applicable	Not applicable		
Subject age & analytical technology											
Childhood											
ELISA	7	498	341	0.93	0.54	1.33	<0.001	85	<0.001	97.2	<0.001
Luminex	3	470	920	−0.03	−0.14	0.08	0.56	0	0.84		
RIAC	1	69	54	1.57	1.16	1.98	<0.001	Not applicable	Not applicable		
Adulthood											
ELISA	4	121	281	0.04	−1.72	1.8	0.97	97	<0.001	Not applicable	Not applicable

ASD: autism spectrum disorder; No.: number; CI: confidence interval; RIAC: recycling immunoaffinity chromatography; ELISA: enzyme-linked immunosorbent assay.

**Table 4:** Meta-regression of peripheral BDNF levels in persons with ASD and healthy controls.

Moderator	No. of comparisions	No. of subjects		Meta-regression				Proportion of variance explained
		ASD	Controls	Slope	95% CI		P-value	R^2^ analog
Age (mean, years)	12	1046	1329	−0.1028	−0.285	0.079	0.237	4.66
Gender (% male)	8	710	1075	0.0628	0.0117	0.114	0.024	58.18
Study design	14	1131	1576	0.248	−1.9765	2.4726	0.812	0
Confounders adjustment	14	1131	1576	−1.8329	−3.992	0.326	0.089	19.18

ASD: autism spectrum disorder; No.: number; CI: confidence interval.
